# When Blood Is Being Difficult: Clotting and Bleeding in a Patient With Antiphospholipid Antibody Syndrome and Supratherapeutic International Normalized Ratio

**DOI:** 10.7759/cureus.25659

**Published:** 2022-06-04

**Authors:** Raghu Tiperneni, Muhammad Tayyeb, Harshil Fichadiya, Ahmad Al-Alwan, Farhan Khalid, Adhithya Rajamohan

**Affiliations:** 1 Internal Medicine, Monmouth Medical Center, Long Branch, USA

**Keywords:** hypercoagulopathy, supratherapeutic, major bleeding events, seronegative antiphospholipid syndrome, deep vein thrombosis (dvt)

## Abstract

Venous thromboembolism includes deep venous thrombosis (DVT) and pulmonary embolism and is the most common cardiovascular disease after coronary artery disease and stroke. Antiphospholipid syndrome (APS) is an autoimmune disorder that is characterized by venous or arterial thrombosis with laboratory evidence of antiphospholipid antibodies. Long-term anticoagulation therapy is required to prevent recurrent DVTs, embolisms, and thrombosis-related complications. Treatment options include vitamin K antagonists, subcutaneous low-molecular-weight heparin, unfractionated heparin, or direct oral anticoagulants. Warfarin (a vitamin K antagonist) remains the mainstay of treatment in APS patients with a prior history of DVT and is associated with elevation of the international normalized ratio which is often used as a marker for therapy appropriateness and warfarin dosing. Here, we describe a unique case of a 65-year-old female with APS on warfarin (given prior lower extremity DVT) presenting with bleeding/hematoma in the left breast and a clot in the left upper extremity.

## Introduction

Deep vein thrombosis (DVT), a type of venous thromboembolism (VTE), is a clot seen in the deep venous system, more common in lower extremities. Although DVTs often cause pain and swelling at the affected site, they can be asymptomatic in up to 70% of cases. VTE occurs for the first time in approximately 100 persons per 100,000 each year in the United States and rises exponentially from fewer than five cases per 100,000 persons among individuals younger than 15 years to approximately 500 cases (0.5%) per 100,000 persons at age 80 years [1]. VTE is often underdiagnosed and a serious, but preventable medical condition. Antiphospholipid syndrome (APS) patients are at an increased risk of developing blood clots. APS is a significant cause of morbidity and mortality and accounts for about 9.5% of DVTs [2]. Primary antithrombotic prophylaxis includes antiplatelets like aspirin, and secondary antithrombotic prophylaxis includes anticoagulation with vitamin k antagonists and direct oral anticoagulants (DOACs) [2]. There are some case reports describing patients with APS having thrombosis and bleeding, but some inciting factors for thrombosis such as norethisterone were noted [3]. However, there are not many cases reported of patients with APS having simultaneous thrombosis and bleeding with an underlying etiology of trauma.

## Case presentation

**Figure 1:**
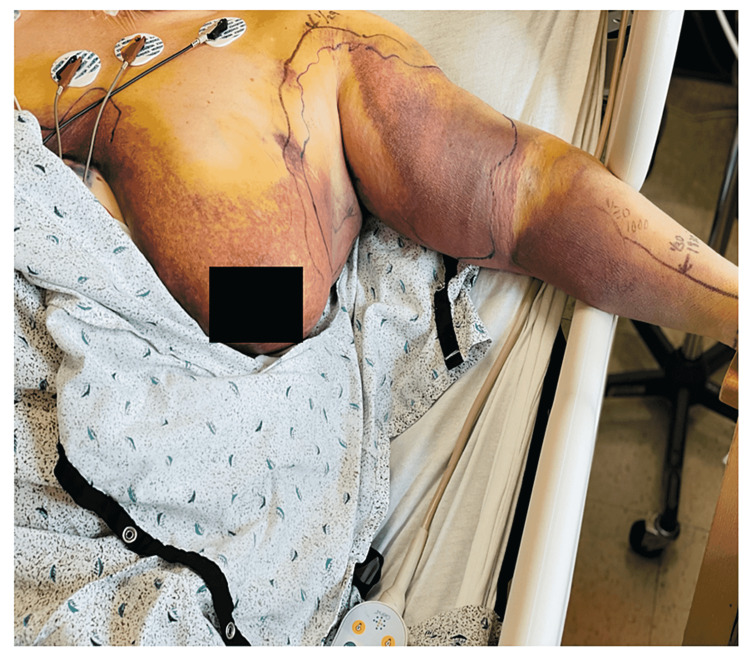
Left breast hematoma and left upper extremity swelling.

A 65-year-old female with a medical history significant for APS with lower extremity DVT in the past (around four years ago) presented to the emergency room for the evaluation of left arm and left breast pain along with swelling for two days after trauma to the left chest wall and left upper arm while lifting boxes at work. Her home medications were reviewed, and she was noted to be on warfarin 5 mg daily for secondary prevention of thrombosis given her previous history of lower extremity DVT. Vital signs were within normal limits. On examination, she had a firm, erythematous, poorly demarcated swelling involving the left breast and upper left arm (Figure 1).

Laboratory tests revealed a hemoglobin level of 10.6 g/dL, a normal platelet count, and an international normalized ratio (INR) of 4.2 (in the supratherapeutic range) (Table 1). Anticoagulation was held on admission due to supratherapeutic INR level. Ultrasound of the left breast revealed a poorly demarcated large 10 × 10 cm hematoma. Imaging with a Doppler ultrasound of the left upper extremity revealed an acute partially occlusive thrombus of the left axillary vein. Overnight, her hemoglobin level dropped from 10.5 g/dL to 6 g/dL without any evidence of external bleeding. At this time, hemodynamic instability was noted with a drop in blood pressure to 90/50 mmHg from 110/70 mmHg (four hours prior) and heart rate at 110 beats per minute (sinus tachycardia on electrocardiogram) with no significant change on the physical examination. A massive transfusion protocol was initiated, and multiple units of packed red blood cells, fresh frozen plasma (FFP), and oral vitamin K were administered (Table 2). Her hemoglobin eventually improved to 9.3 g/dL and INR decreased to 0.9. Subjective improvement in pain, as well as a decrease in the size of the hematoma, were noted. After weighing the risks and benefits and a multidisciplinary discussion, a decision was made to resume anticoagulation with apixaban. She was started on apixaban as an inpatient on day five of hospitalization as her hemoglobin remained stable for two days (since day three, around 9 g/dL) and was closely monitored for signs of bleeding, worsening hematoma, and hemoglobin drop. Her hemoglobin remained stable and showed no evidence of bleeding. She was eventually discharged home on apixaban.

**Table 1:** Pertinent laboratory values during the hospitalization.

Labs	At admission	Day one	Day three
Hemoglobin (g/dL)	10.6	6	9.4
International normalized ratio	4.2	3.7	0.9
Platelets (per microliter of blood)	300,000	310,000	290,000

**Table 2:** Total number of transfusions the patient received during the hospital course.

Transfusions	Total
Packed red blood cells	Four units
Fresh frozen plasma	Two units
Platelets	None

## Discussion

APS is an autoimmune disorder characterized by venous or arterial thrombosis with laboratory evidence of antiphospholipid antibodies [4]. In patients with positive phospholipid antibodies with no history of acute thrombosis, primary thrombosis prevention with aspirin or anticoagulant is usually not recommended based on various trials [5-7]. For non-pregnant patients with APS, warfarin is preferred over DOACs. Recurrent thrombosis despite adequate anticoagulation is uncommon but can occur, as evidenced in our case. It is more common in patients with subtherapeutic INR or therapeutic INR (2-3).

The management of this case posed multiple challenges along the way: (1) addressing bleeding in a patient with an acute clot; (2) resumption of anticoagulation in a patient who had major bleeding while on warfarin.

Unlike warfarin, apixaban does not require frequent monitoring. There is emerging evidence supporting the use of DOACs in patients with APS; however, this is not a universal recommendation and the decision needs to be individualized based on the patient’s characteristics and clinical course. The use of DOACs compared with warfarin is associated with a lower rate of fatal bleeding, the case-fatality rate of major bleeding, cardiovascular mortality, and all-cause mortality, as described in a meta-analysis of randomized clinical trials [8]. Low-molecular-weight heparin is also another reasonable option that is usually considered in APS, where warfarin is not an appropriate option.

In this patient, an upper extremity DVT was possibly precipitated by the trauma. However, due to supratherapeutic INR, this patient also had a simultaneous life-threatening traumatic bleeding manifestation of left breast hematoma. Emergent reversal of the bleeding diathesis with FFP and vitamin K, despite the co-existing thrombosis, as well as hemodynamic stabilization with transfusions, were key elements in the management of this case.

## Conclusions

Asymptomatic individuals with APS (with no history of blood clots or miscarriages) do not require specific treatment; sometimes low-dose aspirin can be considered for patients with high-risk cardiovascular disease (hypertension and diabetes). For the secondary prevention of thrombosis (blood clot), current guidelines suggest patients take an oral anticoagulation (blood thinner) with a target INR of 2-3. Some centers prefer adding low-dose aspirin or increasing the target INR to 3-4 in patients with arterial thrombosis. Emergent reversal of the bleeding diathesis with FFP and vitamin K, as well as hemodynamic stabilization with transfusions, and holding anticoagulation during the acute phase were the key elements in the management of this case with simultaneous bleeding/hematoma and a clot.
